# Impact of exposure to opioids in pregnancy on offspring developmental outcomes in the preschool years: an umbrella review

**DOI:** 10.1136/bmjpo-2024-003058

**Published:** 2025-01-09

**Authors:** Senga Robertson, Thomas Hughes, James Boardman, Alison McFadden, Anne Whittaker, Louise Marryat

**Affiliations:** 1 School of Health Sciences, University of Dundee, Dundee, UK; 2 The University of Edinburgh, Edinburgh, UK; 3 University of Stirling, Stirling, UK

**Keywords:** Child Health, Ophthalmology, Infant, Child Psychiatry, Epidemiology

## Abstract

**Background:**

Early child development sets the course for optimal outcomes across life. Increasing numbers of children worldwide are exposed to opioids in pregnancy and frequently live in environments associated with adverse developmental outcomes. Although multiple systematic reviews have been published in this area, they use different exposures and different types of outcomes. This umbrella review aims to bring together these systematic reviews to provide a comprehensive overview of the evidence around the association between prenatal opioid exposure and preschool developmental outcomes.

**Methods:**

PubMed, MedLine, PsycInfo and Google Scholar were searched up to July 2024. Eligible studies were systematic reviews, meta-analyses or scoping reviews exploring prenatal opioid exposure (illicit opioids and prescribed treatments for opioid dependence) and developmental outcomes up to age 5. Reviews were screened by two authors. Quality assessment was undertaken using the Joanna Briggs Institute checklist for umbrella reviews. Degree of overlap was examined. Due to heterogeneity within the sample, no meta-analyses were undertaken and results were synthesised narratively.

**Results:**

11 reviews were included containing 478 individual papers. The overlap was slight (corrected cover area=5%). Developmental outcomes associated with prenatal opioid exposure included visual function, motor skills, externalising problems and language difficulties. No conclusive evidence was available for cognitive development or internalising symptoms. In cognitive, and motor, skills, findings differed by age, with later preschool findings being weaker. Authors frequently highlighted issues with poor quality research in the original studies, including small sample sizes and lack of controlling for confounding.

**Conclusions:**

Multiple areas of child development were associated with prenatal opioid exposure; however, evidence was weak. Robust research, with larger sample sizes and adequate accounting for confounding, is needed to provide accurate information for women of childbearing age and practitioners to guide policy and ensure that appropriate funding, support and follow-up are in place.

**PROSPERO registration number:**

CRD42022307992.

**WHAT IS ALREADY KNOWN ON THIS TOPIC**

- Evidence indicates that prenatal opioid exposure is associated with a range of adverse outcomes in childhood.

**WHAT THIS STUDY ADDS**

- This study highlighted relatively robust evidence of associations between prenatal opioid exposure and visual function, motor skills, externalising problems and language difficulties; however, the overall quality of evidence was weak, with small sample sizes and lack of controlling for confounding frequently apparent.
- No conclusive evidence was available for an association between prenatal opioid exposure and cognitive development or internalising symptoms; however, this may be due to more formal testing not occurring until later.

**HOW THIS STUDY MIGHT AFFECT RESEARCH, PRACTICE OR POLICY**

- This evidence can enable health, social care and education professionals working with children exposed to opioids in pregnancy to more closely monitor these children, and for additional support to be put in place where required for example, around visual function, motor skills, externalising problems and language difficulties. Service providers should invest in universal health provision to ensure that these groups are properly supported with more targeted intervention where required.
- This review highlighted the lack of robust evidence about the long-term impacts of prenatal opioid exposure: further, and more robust, research, adequately controlling for confounding, and with longer term follow-up, is, therefore, required across domains.

## Introduction

 As the world experiences an opioid crisis, increasing numbers of children are being exposed to opioids during pregnancy.^1^ Substantial research has explored the impact of exposure to opioids on birth and neonatal outcomes. Findings demonstrate several adverse impacts, including lower birth weight, smaller head circumference, atypical brain development, shorter length and higher rates of preterm birth and infant death.^2 3^ Until recently, there has been less focus on longer term outcomes for children, primarily due to methodical challenges in following up this population using traditional methods, such as longitudinal cohort studies, as was demonstrated in Sim *et al*’s article.^4^ The evidence available highlights later adverse outcomes across a range of domains, including education, behavioural difficulties, social and health outcomes.^5^,^8^ These relatively recent advances are leading to changes in the way that practitioners care for affected families; however, more research is needed to support further improvements to service provision.^9^

The early years and preschool period (considered here as birth to 5 years) is a critical period of development: it sets the course for a wide range of mental and physical health, as well as educational and social outcomes throughout life.^10 11^ It is a time when parents generally have the most influence on children’s outcomes, before they are impacted more heavily by schools and peers.^12^ For both reasons, it is a period of key interest to researchers and policymakers alike (eg,^13^), who are keen to reveal the mechanisms influencing differences in outcomes, and thus discover potential areas for early intervention. Poor early child development is associated with a range of adverse childhood experiences, including living in poverty, low parental education levels, child neglect and/or maltreatment, domestic abuse, parental mental-health issues, and removal from the birth parent into social care.^14^ Children with parents who use drugs are at high risk of experiencing many of these events.^15^ Brain scans of infants exposed to opioids in pregnancy taken shortly after birth have, additionally, indicated differences in major white matter tracts, which are independent of head size.^2^ Previous research in the general population found that structural brain development in children mediates the relationship between poverty and academic achievement.^16^ Further complexities lie in the relationship between genetic and environmental influences, whereby vulnerability to neurodevelopmental disorders, neonatal abstinence syndrome and future substance use are associated with a complex interaction of genetic and environmental factors.^6^ However, research is currently disparate, and a more precise characterisation of such difficulties for children exposed to opioids in pregnancy is required. In recent years, several systematic reviews and meta-analyses focusing on different opioid exposures (eg, methadone/buprenorphine/illicit opioids/neonatal abstinence syndrome (more recently known as neonatal opioid withdrawal syndrome (NOWS)) and different groups of developmental outcomes (eg, vision/cognitive development/internalising and externalising behaviours) have been published. To date, however, there has been no synthesis of such findings, resulting in a lack of overview of evidence around the impact of opioid exposure on child development across these different groups of developmental outcomes in the preschool period. This umbrella review (systematic review of reviews) aims to bring together the current evidence on prenatal opioid exposure (focusing on illicit opioids and/or opioid-based treatment for substance use disorders, not solely including intermittent use of opioids and/or opioid exposure through chronic pain relief medication) and social, emotional, behavioural, cognitive and visual developmental outcomes. The aim is to provide a comprehensive overview of the available evidence exploring prenatal opioid exposure and preschool developmental outcomes up to age 5. This will provide information for health, social care and education professionals working in this area, as well as highlighting areas for future research in the field. By focusing on evidence in the 0–5 period, there is the potential for this to lead to interventions based early in children’s life, which have been evidenced to have the largest impacts, in the school years and beyond.^10^

## Methods

The aim of this umbrella review was to synthesise the evidence identifying the impact of exposure to opioids in pregnancy across child developmental outcomes in the preschool years. As several systematic reviews covering different aspects of child developmental outcomes in various settings were identified in a scoping search of the literature, it was decided that an umbrella review (ie, a systematic review of reviews) would be conducted to bring together these different elements into a cohesive exploration of the impact of in utero opioid exposure on preschool developmental outcomes. The review was conducted in accordance with the Joanna Briggs Institute’s (JBI) Manual for Umbrella Reviews^17^ and followed the Preferred Reporting Item for Systematic Review and Meta-Analysis (PRISMA) statement (online supplemental file 1).^18^

### Search strategy

A systematic review protocol was developed and registered in advance with the International Prospective Register of Systematic Reviews (PROSPERO) on the 1 February 2022 (CRD42022307992).

Reviews were identified by searching electronic databases PubMed (2016–current), MedLine (2016–current), PsycInfo (2016–current) and Google Scholar (first 100 hits) to identify literature reviews (including systematic reviews, meta-analyses and scoping reviews) published up until the search was run on 28 August 2023, with an update to the search run on 2 July 2024. The search strategy was developed using the population/problem/patient, intervention/issue and outcome framework alongside a restriction in the type of study design. Through this, a search strategy was created based on four blocks of keywords: (a) the population of mothers; (b) the exposure of opioids; (c) the developmental outcomes and (d) the study design (table 1). Full search strategies can be found in online supplemental file 2.

**Table 1:** Search strategy

Domain	Key terms
Population	“pregnancy” OR “mother” OR “prenatal” (and synonyms)
Intervention	“Opiates” OR “Opioids” OR “heroin” OR “methadone” or “buprenorphine” OR “morphine”
Outcome	“development” OR “developmental outcomes” OR “cognitive” OR “ emotional” or “behavio*ral”IN “child*”
Study design	“Meta-analysis” OR “systematic review” OR “literature review”

### Inclusion and exclusion criteria

Papers were eligible for inclusion if they were systematic reviews or meta-analyses of observational studies (ie, cross sectional, cohort—retrospective/prospective and case–control) and/or intervention studies (eg, RCTs), which (a) examined the impacts of in utero exposure to opioids during pregnancy and (b) related these to child cognitive, social, emotional, behavioural and visual development at ages 0–5 years (or contained results, which could be restricted to these ages) (to include preschool age children across different country settings) in a high-income country (HIC) as defined by the World Bank.^19^ Full inclusion and exclusion criteria are presented in table 2. Articles were excluded if exposure to opioids in pregnancy was not reported and examined; the outcome was not developmental in nature or in preschool aged children; they were commentary, editorial or primary studies; and were not human in nature (where reviews of human and animal research were carried out in separate searches and results separated within the same paper this was included).

**Table 2:** Full inclusion and exclusion criteria

Characteristic	Inclusion criteria	Exclusion criteria
Population	Studies involving children aged 0–5 years.	Studies involving children with outcomes over the age of 5 years.Studies focusing on non-human subjects eg, mice.
Intervention/initial assessment	Exposure to illicit opioids/prescription opioids for treatment of addiction in pregnancy	Studies in general population or focusing on polydrug use/other substance use. Studies with prescription opioids for pain relief.
Outcomes	Any outcome relating to behavioural, cognitive or developmental outcomes.	Studies focusing on NOWS (as an outcome rather than a proxy for exposure) or other physical health outcomes.
Study design	Meta-analysis or reviews.	Empirical studies, reports containing no new data, such as editorials
Location	HICs as defined by the World Bank	Low- and middle-income countries
Language	English	Languages other than English
Date	Up to 2 July 2024 (second search)	

HICshigh-income countriesNOWSneonatal opioid withdrawal syndrome

### Data screening

Search results were screened within Covidence to identify reviews meeting inclusion criteria as specified above. Screening took place in two phases—titles and abstracts, and full text. Articles were screened independently based on titles and abstracts by two researchers (LM and TH/SR). All conflicts were reviewed again and resolved through a consensus. The full texts of the articles were then independently scrutinised by both researchers, and conflicts were resolved by consensus.

### Quality assessment

The quality of the included articles was assessed using the JBI critical appraisal tool for umbrella reviews.^17^ In line with the JBI guidance, the authors agreed in advance that the low-quality reviews were defined as meeting fewer than a third of the criteria, medium-quality reviews met between a third to two-thirds of the criteria and high-quality reviews met between two-thirds to all of the criteria in the appraisal tool. We chose not to exclude reviews based on their quality, as the findings still held some relevance. Rather, where reviews were deemed to be of low quality, this is highlighted in the Results and Discussion sections.

### Data extraction

Data extraction was performed by one reviewer (TH, LM or SR), and then checked by one other reviewer. Any discrepancies were resolved through discussion. The JBI template for data extraction was used. This recorded information for each study regarding: author/year of publication, objectives of the study, participants, setting, phenomena of interest, method of analysis/study design, outcomes assessed, results, heterogeneity and conclusions.

### Overlap

The degree of overlap of the included primary studies was examined from all reviews in our umbrella review, using the corrected cover area (CCA). The CCA was calculated as a measure of overlap and described as a value indicating the proportion and percentage of overlap.^20^ These were calculated as follows (N=number of citations, including double counting, r=number of publications and c=number of reviews included in the umbrella review)

$$CA\left(covered\;area\right)\;=\frac{N}{rc}$$

$$CCA\;\left(Corrected\;CA\right)\;=\;\frac{N-r}{rc-r}$$

### Data synthesis

Characteristics and findings of the included systematic reviews and meta-analyses are presented in tables, summarising the population, timeframe, exposures and main findings.

### Public and patient involvement (PPI)

The research team carried out PPI with women via relevant charities and clinicians supporting women who use substances in pregnancy and parenting. The PPI work involved discussing research plans and findings. Feedback influenced the way results were discussed in this article. The cocreation of dissemination materials stemming from the findings for (and with) women who use opioids is planned.

## Results

**Figure 1:**
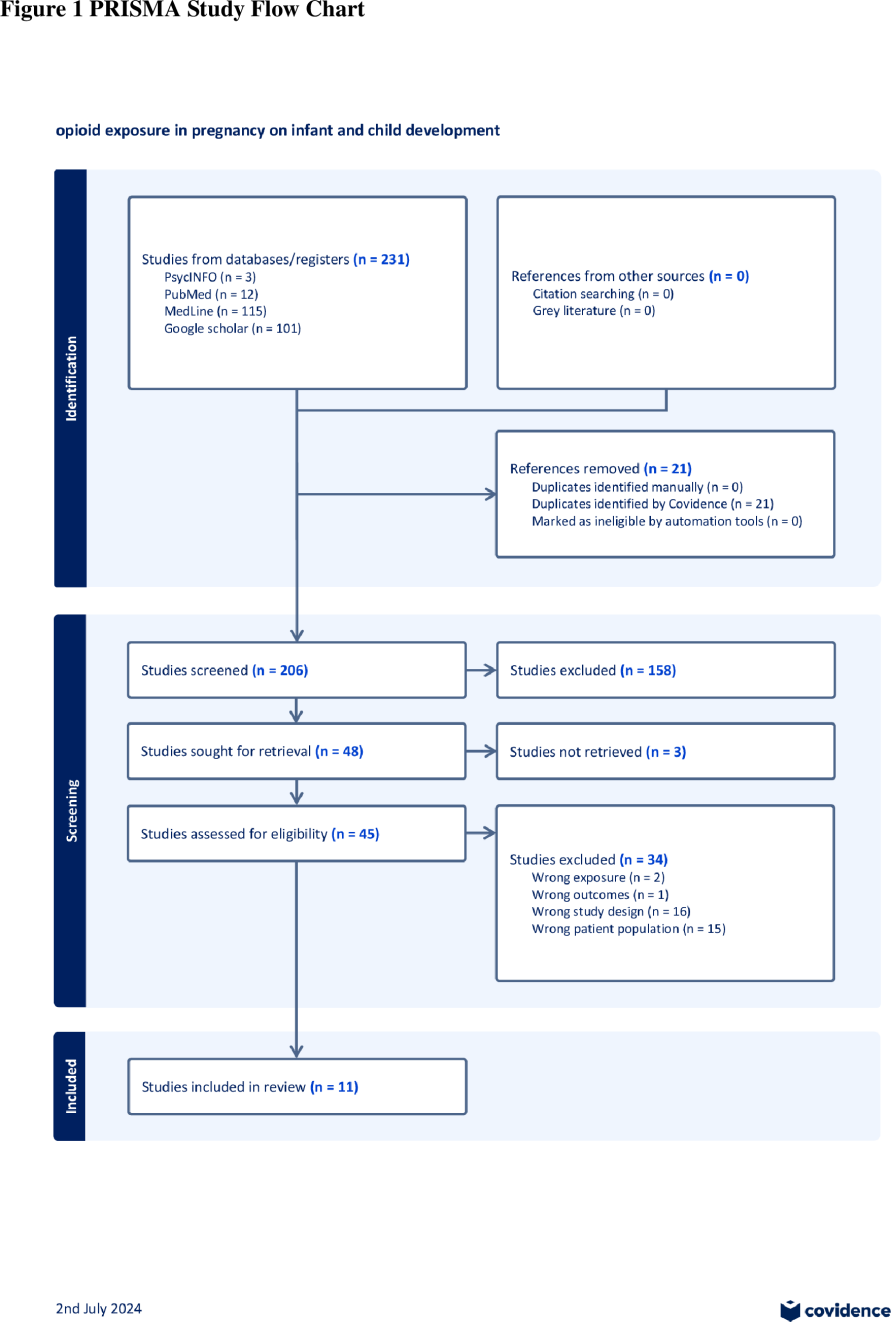
PRISMA flowchart. It displays the PRISMA flowchart for the umbrella review. PRISMA, preferred reporting item for systematic review and meta-analysis.

Figure 1 displays the PRISMA flowchart that demonstrates the selection of reviews. Overall, 231 records were identified from database searches, and 21 duplicates were removed from these. A total of 206 titles and abstracts were screened for eligibility, following which 48 articles were selected for full-text review (3 of which the full texts were unavailable, despite efforts to locate them). 34 were subsequently excluded. Reasons for exclusion at this stage included wrong exposures (reviews focusing on polydrug use) (2); did not address developmental outcomes (1); wrong study design (eg, narrative reviews) (16) and wrong patient population (eg, focus on later child outcomes and/or maternal outcomes/experiences) (15). Overall, 11 reviews met the inclusion criteria for this umbrella review. Of these, 7 reported a meta-analysis for at least some outcomes.

### Degree of overlap

There were 710 citations in total from a pool of 478 papers: 110 papers were cited in more than one review, with 368 being featured in one review only. Covered Area (CA) was determined to be 13.7% and CCA to be 5%. This indicates that the level of overlap was considered to be slight and did not need to be corrected for CCA (0–5=slight; 6–10=moderate; 11–15=high and over 15=very high).^20^

### Critical appraisal

When the systematic reviews were assessed for quality against the critical appraisal tools from the JBI, 8 (out of 11) included reviews were classified as high quality, 2 reviews were of moderate quality and 1 was considered low quality (table 3). All reviews had clearly set out a research question or aim and almost all reviews had appropriate inclusion criteria. Search and appraisal methods were generally less clearly defined, with only three reviews confirming that two or more reviewers conducted the critical appraisal, and only four discussed methods to minimise errors in data extraction, such as having more than one person extracting data. Where recommendations for policy and practice and/or research were made, these were all based on data presented.

**Table 3:** Quality appraisal results

	Clear question	Appropriate inclusion criteria	Appropriate search strategy	Adequate resource	Appropriate appraisal	2+reviewers appraising	Methods to minimise errors in extraction	Methods to combine appropriate	Publication bias assessed	Policy or practice recommendations supported	Future research appropriate	Overall rating
Andersen *et al*^21^	Y	Y	Y	Y	Y	N	U	Y	Y	Y	Y	High
Arter *et al*^22^	Y	Y	N	Y	Y	Y	Y	Y	N	Y	N/A	High
Conradt *et al*^23^	Y	Y	Y	U	N	U	U	N/A	N	N/A	Y	Medium
Hemmati *et al*^31^	Y	Y	Y	U	Y	U	U	Y	Y	Y	Y	High
Lee *et al*^24^	Y	Y	Y	Y	Y	U	U	Y	Y	Y	Y	High
Monnelly *et al*^2^	Y	Y	Y	Y	Y	Y	Y	Y	N	Y	Y	High
Nelson *et al*^26^	Y	Y	Y	Y	Y	Y	Y	Y	Y	Y	N/A	High
Rees *et al*^27^	Y	Y	Y	Y	U	U	Y	Y	N	Y	N/A	High
Romanowicz *et al*^28^	Y	Y	Y	Y	N/A	N	U	Y	N	Y	Y	Medium
Welton *et al*^29^	Y	U	U	U	Y	U	U	N	N	Y	N/A	Low
Yeoh *et al*^30^	Y	Y	Y	Y	Y	N	U	Y	Y	N/A	Y	High

N/ANot Applicable

### Study characteristics

Table 4 summarises the findings of the included reviews (full data extraction tables can be found in online supplemental file 3). A range of developmental outcomes were assessed across 11 reviews, including the overall development (n=2), cognitive skills and language development (n=10), motor skills (n=5), externalising problems (ie, behavioural problems and attention/hyperactivity problems) (n=8), internalising problems (ie, emotional difficulties) (n=4) and visual outcomes (n=5). The following sections explore each of these sets of results in further detail. Aside from one paper, which focused exclusively on visual outcomes following opioid exposure, the majority explored more than one type of outcome.

**Table 4:** Summary of findings of included systematic reviews

	No. of studies included	Meta-analysis	Exposure	Age of children	Composite development	Cognitive skills and language development	Motor skills	Externalising problems	Internalising problems	Visual outcomes
Andersen *et al*^21^	29	Yes	Methadone or buprenorphine	3 m+*	+	+	+	+		+
Arter *et al*^22^	43	No	Illicit or prescription opioids	2 years +*	~	~	~	~	+	
Conradt *et al*^23^	52	No	Prenatal opioid exposure	0–18*		~		+		+
Hemmati *et al*^31^	9	No	Prenatal opioid exposure	0–18*						+
Lee *et al*^24^	16	Yes	Opioid dependency	0–12*		+		+	+	
Monnelly *et al*^2^	41	Yes	Methadone	0–18*		~	~	+		~
Nelson *et al*^26^	27	Yes	At least 2 months of prenatal MAT	1–60 m		~				
Rees *et al*^27^	15	Yes	Neonatal abstinence syndrome	0–16*		+		+	~	+
Romanowicz *et al*^28^	12	No	Parental opioid use	0–16*		+		~		
Welton *et al*^29^	19	No	Opioid use (excluding polydrug use/alcohol)	2+*		~	~	+		
Yeoh *et al*^30^	26	Yes	Prenatal opioid exposure	0–16*		+	+		~	

+, association found; −, no association found; ~, mixed/inconclusive.

* Results were extracted up to 6 years only.

mmonthsMATmedication-assisted treatment

### Overall development

Two reviews^21 22^ produced data on the overall developmental outcomes following prenatal exposure to opioids. Andersen *et al* found a positive relationship between methadone and buprenorphine exposure in pregnancy, and overall cognitive, psychomotor, behavioural, attentional and executive functioning (overall effect size (ES)=0.49, 95% CI 0.38 to 0.59),^21^ but results in Arter *et al*’s article were more mixed, with five of the eight studies finding a positive association, but the rest showing no significant difference.^22^ Arter *et al's* review hypothesised that the mixed results in their review were due to the version of the Bayley scales being used, with papers using the newer scales more consistently showing an association.^22^

### Cognitive skills and language development

Ten reviews explored outcomes relating to cognitive and/or language development in the preschool period: ten looked at cognitive outcomes^21^,^30^ and six looked at language development.^22^,^2527 29^

Results for cognitive development were mixed: five reviews^21 24 27 28 30^ found consistent results in terms of a positive association between opioid exposure in pregnancy and poorer cognitive outcomes. It should be noted that Romanowicz *et al*^28^ and Rees *et al*^27^ only included one study in this result, respectively. Andersen^21^ and Lee^24^ both found that opioid-exposed children had significantly lower cognition scores than non-exposed infants (d=0.77, 95% CI –1.06 to –0.48). This finding was replicated in Yeoh *et al*’s article,^30^ but this review also broke down results by age band and found a stronger effect in infancy (0–24 months) (d= −0.52, 95% CI −0.74 to −0.31; p<0.001) than in preschoolers (age 3–6) (d = −0.38, 95% CI −0.69 to −0.07; p<0.02), compared with non-exposed controls. Similarly, Monnelly *et al*, although finding no significant difference between children exposed to methadone versus unexposed controls in terms of cognitive outcomes in infancy (6 months), found a significant weighted mean difference at age 2 (d=−4.3, 95% CI −7.24 to –1.63), but then no difference beyond this point.^25^ The remaining reviews were inconclusive: three^23 26 29^ initially found an association between opioid exposure and poorer cognitive outcomes; however, results were non-significant once various confounding factors were controlled for, including socioeconomic factors^23 29^ and prenatal tobacco exposure.^26^ Andersen *et al* reported no difference in cognitive development between children exposed to opioids through medication-assisted treatment, compared with those exposed through illicit opioids (based on five studies).^21^ Studies within the reviews were highly heterogeneous in the measurement of cognitive development: the vast majority of studies used the Bayley scales; however, multiple other measures were used. In Anderson *et al*’s review alone, 27 different measurement scales were used in assessing cognitive and motor functioning.^21^

Of the six reviews focusing on language development, five found that children exposed to opioids had poorer language skills in the preschool period.^23^,^2527 29^ Lee *et al* looked at expressive and receptive language scores, respectively, and found associations between opioid exposure and poorer performance on both: expressive language scores (d=−0.65, 95% CI –0.97 to –0.34) and receptive language scores (d=−0.74, 95% CI –1.12 to –0.36).^24^ Just one review found mixed results; however, four out of five primary studies reviewed in this article did find a significant association between opioid exposure and language skills.^22^ In addition, Conradt *et al* highlighted one Randomised Control Trial (RCT), which found that children exposed to buprenorphine (compared with methadone) had poorer language skills at 12 months.^23^

### Motor skills

Different aspects of motor skills were captured in five reviews^21 22 25 29 30^: these included motor development (ie, the capability in movement that usually occurs as children age), with some reviews looking more specifically at gross motor skills (movements using large muscle groups eg, walking) and/or fine motor skills (requiring more precise movement eg, writing). In addition, some reviews captured psychomotor skills, which are the skills developed from learning a new motor skill and bring together cognitive and movement skills. Results consistently found that motor skills overall were poorer in children exposed to opioids than unexposed children in the preschool period^21 22 25 29 30^ for example, Andersen *et al* found an ES of 0.56 (95% CI 0.28 to 0.85).^21^ Although most studies used parental measures of motor skills, one study also used observed motor skills and found similar results, although at a slightly weaker level (eg, ES=0.37, 95% CI 0.19 to 0.55).^21^ Welton *et al*’s review revealed inconsistent results in terms of motor difficulties resolving with maturity: two studies within their review indicated that motor delay normalised by ages 2–3 years, and another study reported below normal development remaining at age 5–6 years.^29^ No differences were found between children exposed to illicit versus prescription (eg, methadone/buprenorphine) opioids or between exposed children who were removed or not removed from the birth mother.^30^ Evidence around fine motor skills was inconclusive, with one review finding no relationship once confounding factors were controlled for.^27^ In relation to psychomotor skills, results consistently found that children exposed to opioids had poorer psychomotor skills than unexposed children.^21 25^

### Externalising problems

Externalising problems (ie, behavioural problems and attention/hyperactivity problems) were reported in eight reviews; all found an overall positive relationship between exposure to opioids in pregnancy and behavioural problems (including conduct disorder and symptoms, such as aggression).^21^,^2527^ Five reviews found opioid exposure to be related to attention difficulties and/or hyperactivity, including attention deficit hyperactivity disorder (ADHD).^21^,^2429^ These results were seen to hold true after controlling for confounding factors, including socioeconomic status, sex and age^23^; indeed, Welton *et al* noted that effects appeared to be exacerbated for children living in low socio-economic classification households and when children were male.^29^ Another review, however, found inconsistent results when exploring the relationship between NOWS and ADHD.^27^ In addition, Romanowicz *et al* found a significant relationship between methadone and hyperactivity, compared with unexposed children, but no relationship with focused attention.^28^

### Internalising problems

Results for internalising symptoms (ie, emotional difficulties, such as somatic complaints and anxiety) were rarely explored in the selected reviews, and results were inconclusive. Two reviews found an association between opioid exposure and internalising symptoms^23 24^; however, further two reviews found inconsistent results.^22 27^

### Visual outcomes

Five reviews addressed the impact of opioid exposure on optical development.^21 23 25 27 31^ All found opioid exposure to be associated with at least one measure of visual outcomes in preschoolers. Specifically, three reviews found that children exposed to opioids had lower visual motor/perceptual performance scores, poorer lower left visual eye acuity and increased reports of nystagmus (rhythmic involuntary oscillations of the eyes) and strabismus (commonly known as a ‘squint’).^23 25 27^ Hemmati *et al* highlighted the relative risk of ophthalmic abnormalities as 5.1 (95% CI 1.3 to 20; p=0.02).^31^ Monnelly *et al*, however, noted that the majority of studies within their review, which showed positive visual outcome results, were of poor quality.^25^ By contrast, one review found no difference in visual perception,^23^ and Monnelly *et al* and Hemmati *et al* found mixed results for visual evoked potentials,^25 31^ with one study finding no difference between exposed children and controls at 3 years, although other studies found significant differences at 4–6 months.

## Discussion

This study was the first, to our knowledge, to bring together systematic reviews exploring the impact of opioids on different aspects of early child development, thus giving a comprehensive overview of the evidence across developmental domains. The 11 reviews included in the umbrella review covered multiple domains of early child development that have been studied to date, including cognitive, language, motor, internalising, externalising and visual development. Some of these fields have clearly had more research into them than others: cognitive development and externalising problems were covered within the majority of reviews, while aspects, such as internalising problems, were included in fewer reviews. It appears that this largely reflects the amount of primary research being conducted in these fields.

There appeared to be fairly strong evidence of an association between exposure to opioids and visual outcomes, motor skills, externalising problems and language difficulties, with an association between prenatal opioid exposure and adverse outcomes demonstrated in the majority of studies. Even here though, review authors repeatedly noted methodological issues in studies in this field, primarily due to very small sample sizes in the empirical studies and/or a lack of control group/controlling for confounding. Indeed, at times, when confounding factors were adequately controlled for, associations were seen to become non-significant (eg,^25^ and ^29^). Additionally, it should be noted that some of the inconsistency in findings for diagnostic aspects, such as ADHD, may result from the young age at which children appearing in this study are being assessed, with clinicians in many areas reluctant to make a diagnosis at this young age.^32^

Other areas of development produced more inconclusive results: this included cognitive skills and internalising symptoms. In terms of cognitive development, there appeared to be variation in results depending on the age of the children, with stronger effects in infancy than in the later preschool period. It could be that, although these children have delayed development initially, they can catch-up with their peers by age 3, either naturally or potentially with the support of preschool care settings and/or family interventions. This is an important observation about resilience that focuses research attention on understanding better ways to support opioid-exposed children in the preschool years. Evidence about the impact of preschool interventions on early child development is inconclusive and primarily based on weak evidence,^33^ and there is limited evidence on the impact of such interventions for children whose parents use drugs.^34 35^ Findings around cognitive skills contrast, however, with the more conclusive findings around language difficulties, which demonstrated an association between prenatal opioid exposure and both receptive and expressive language difficulties. Other studies have shown language skills to be closely related to cognitive development and later educational achievement; thus, the results here may be related to the aspects of non-verbal cognitive functioning measured in these studies.^36^ Additionally, the lack of evidence in later stages could be due to methodological issues in the samples, such as attrition over time and challenges in following up children at later ages resulting in smaller sample sizes.^4^

Evidence on internalising symptoms was also very mixed. Internalising symptoms in the preschool years are often measured by non-specific symptoms, such as frequent stomachaches or headaches, which can be difficult to identify or distinguish from normative behaviour at this stage.^37^ Research on trajectories of internalising behaviours in a general population also indicate that these rise substantially in later childhood, and particularly in adolescence^38 39^; it could be that the preschool period is too early to detect such differences, which may emerge later. A recent systematic review and meta-analysis exploring internalising outcomes at 0–18 years following any parental substance use found that either fathers’ substance use or mothers’ substance use had an independent association with internalising symptoms (fathers: OR=1.42, 95% CI 1.12 to 1.81; mothers: OR = 1.60, 95% CI 1.25 to 2.06).^7^ The similarity in these results suggests that these findings may be linked to parenting and the environment, rather than exposure to substances during pregnancy.

Across all domains, the lack of appropriate controlling for confounding factors within studies has been noted. This is important because child development in the early years is not only associated with prenatal exposures, but with a range of other factors, many of which are also associated with maternal substance use. These include maternal factors (eg, education), genetic factors (eg, in relation to ADHD), household factors (eg, poverty, exposure to domestic abuse and the home learning environment) and individual factors (eg, low birth weight).^14^,^16^ Adequately controlling for confounding in these studies must be a key priority in future research in this area.

In many HICs, such as the UK, where not insignificant numbers of children are exposed to opioids in pregnancy each year, there is no targeted follow-up of this specific group of children beyond the postpartum period. While routine follow-up, such as the Universal Health Visiting Pathway in Scotland, will identify many of these issues, evidence indicates that children from more disadvantaged areas are less likely to receive such reviews, especially beyond the postpartum period.^40^ In addition, where issues are identified through childhood home visiting programmes, research suggests that referrals are often not successfully followed-through.^41^ In domains, where the evidence is robust and where early intervention can make a substantial difference to long-term outcomes, such as strabismus, there is a question as to whether these children should be part of a more targeted pathway. A key recommendation from this umbrella review is, therefore, that additional resource should be made available to universal services, such as health visiting, to ensure that these ‘hard to engage’ families are offered better routine follow-up and targeted additional support when needed.

### Strengths and limitations

This review used a robust and thorough search strategy to bring together the most comprehensive review of evidence relating to the impact of opioid exposure in pregnancy on the overall child development. An overlap assessment was carried out to ensure that the results from a small range of studies were not being overamplified. At least two authors were involved in each stage of the screening, appraisal and data extraction processes. 11 systematic reviews were identified, comprising results from 478 individual papers. Quality appraisal of the reviews indicated that the systematic reviews were of high quality; however, the evidence that they were based on was often reported to be limited. This was related to a lack of robust long-term follow-up and controlling for confounding factors. Studies in this area are often challenging to conduct using traditional research methods; substance use is under-reported in whole population studies,^42^ and when families are captured in initial data, they often have highly complex lives, with a large proportion of children spending time living away from the birth parents, making them difficult to track over longer periods of time and subsequently limiting outcome data.^4^

This umbrella review focused on systematic reviews and meta-analyses, and thus may have missed empirical studies not captured by systematic reviews within the timeframe of the reviews. As this is a constantly evolving field, this may mean that some more recent research has been missed. The study was restricted to HICs in order to bring together more homogenous findings, which may mean that systematic reviews focusing on low- and middle-income countries have been missed. There was a high level of heterogeneity between the data, both in terms of exposures and outcomes being measured; for this reason, no further meta-analysis was conducted.

## Conclusions

This review of reviews highlighted several areas of child development where there appear to be differences in outcomes for children exposed to opioids in pregnancy, which are apparent before the child reaches school, including visual outcomes, motor skills, externalising problems and language difficulties. Having the evidence on the impact of exposure to opioids in pregnancy on child developmental outcomes is important for health and social work practitioners, as well as women who used opioids, to enable them to understand the likely impact of opioids on the child. This evidence also enables children to be more closely monitored, with additional support put in place where required. In many areas, however, evidence is still weak, with small numbers and lack of controlling for confounding, which is critical in relation to child developmental outcomes, which are substantially impacted by the child’s environment.^43^ In some areas, such as internalising behaviours and cognitive development, the evidence in the preschool period remains very mixed, with no conclusions able to be reached. The increasingly sophisticated research enabled by population-level administrative data infrastructure will help to develop the evidence base in this field, particularly where multicountry data can be pooled.

## supplementary material

10.1136/bmjpo-2024-003058 online supplemental file 1

10.1136/bmjpo-2024-003058 online supplemental file 2

10.1136/bmjpo-2024-003058 online supplemental file 3

## Data Availability

All data relevant to the study are included in the article or uploaded as supplementary information.
