# Supplementary material for: Impact of exposure to opioids in pregnancy on offspring developmental outcomes in the preschool years: an umbrella review
Source: BMJ Paediatr Open. 2025 Jan 9;9(1):e003058. doi: 10.1136/bmjpo-2024-003058 (PMC11749434; doi:10.1136/bmjpo-2024-003058)
Supplement: online supplemental file 2 [file bmjpo-9-1-s002.pdf]

Supplementary File 2: Search Strategy

Title and Abstract screening: PubMed (2016 - current), MedLine (2016 - current) and PsychInfo (2016 - current) and Google Scholar (2016 – current)(1st 100 hits screened).

Systematic reviews, Meta-analyses, Scoping Reviews

PubMed:

((((mother[Title/Abstract] OR pregnan*[Title/Abstract] OR prenatal[Title/Abstract]) AND (opioid[Title/Abstract] OR opiate[Title/Abstract] OR heroin[Title/Abstract] OR buprenorphine[Title/Abstract] OR methadone[Title/Abstract] OR morphine[Title/Abstract])) AND (development[Title/Abstract] OR developmental[Title/Abstract] OR cognitive[Title/Abstract] OR emotional[Title/Abstract] OR behavioural[Title/Abstract] OR behaviour[Title/Abstract])) AND (systematic review[Title/Abstract] OR meta analysis[Title/Abstract] OR scoping[Title/Abstract] OR literature[Title/Abstract])) AND (child[Title/Abstract])

MedLine

MESH terms:

Analgesics, Opioid AND Pregnancy AND (Child development OR Child behavior)

PsychInfo

(((abstract: (methadone)) OR ((abstract: (buprenorphine)) OR ((abstract: (morphine))) OR (abstract: (opioid)) OR (abstract: (opiate)) OR (abstract: (heroin))) AND ((abstract: (mother)) OR (abstract: (pregnan*)) OR (abstract: (prenatal))) AND ((abstract: (emotion*)) OR (abstract: (behaviour*)) OR (abstract: (development)) OR (abstract: (cognitive)) OR (abstract: (behavior*))) AND (Year: [2016 TO 2024]) AND Methodology: Literature Review)

Google scholar – 1st 100 results

(opioid) AND ((pregnan*) OR (mother*)) AND (child development) AND (systematic review)
