# Supplementary material for: Impact of exposure to opioids in pregnancy on offspring developmental outcomes in the preschool years: an umbrella review
Source: BMJ Paediatr Open. 2025 Jan 9;9(1):e003058. doi: 10.1136/bmjpo-2024-003058 (PMC11749434; doi:10.1136/bmjpo-2024-003058)
Supplement: online supplemental file 3 [file bmjpo-9-1-s003.pdf]

Author/ year/ title	Objectives	Included studies	Setting	Meta-analysis	Results	Heterogeneity	Limitations	Quality Score
**Andersen et al., 2020 Prenatal exposure to methadone or buprenorphine and long-term outcomes: A meta-analysis.**	To review evidence on long-term cognitive, psychomotor, behavioural, attentional, executive and visual outcomes for children prenatally exposed to methadone or buprenorphine	Inclusion criteria: evidence of cognitive, psychomotor, behavioural, attentional, executive and visual outcomes I children exposed to methadone or buprenorphine; children aged 3m+; control group available. 29 studies to August 2018.	Global (language = English or Scandinavian)	Yes	**Overall development.** Small but significant difference in overall outcomes between children exposed to methadone/buprenorphine and those not in terms of overall cognitive, psychomotor, behavioural, attentional and executive functioning (overall effect size (ES)= 0.49, 95% CI, 0.38 to 0.59).<br>**Cognitive skills and language development.** 20 studies looked at cognitive outcomes (19 in children under 6). Exposed children more likely to have poorer cognitive outcomes than unexposed (ES= 0.56, 95% CI, 0.38 to 0.74). There was no statistically significant difference in cognitive outcomes in 5 studies however when children exposed to opioids through MAT were compared with children exposed through illicit opioids.<br>**Motor skills.** 12 studies looked at psychomotor outcomes (11 in children under 6): exposed children more likely to have poorer psychomotor skills than unexposed (ES= 0.56, 95% CI, 0.28 to 0.85). 3 of these studies explored differences between children exposed to opioids through MAT and children exposed through illicit opioids: no statistically significant difference was found. 3 studies contained observed measures of motor activity: exposed children more also more likely to have observed poorer motor skills than unexposed (ES= 0.37, 95% CI, 0.19 to 0.55).<br>**Externalising problems.** 7 studies looked at behaviour (6 in children under 6) and concluded that exposed children had higher levels of behavioural problems (ES= 0.52, 95% CI, 0.27 to 0.77). 7 studies also looked at attention and executive functioning: exposed children had higher levels of difficulties in this area (ES= 0.40, 95% CI, 0.19 to 0.61).<br>**Visual outcomes.** 5 studies explored visual outcomes: exposed children had higher levels of visual problems than unexposed (ES= 0.25, 95% CI, 0.01 to 0.50).	Strong heterogeneity. Large methodological differences in measures used, age at outcome.	Low number of studies and limited quality. Some cohorts had high levels of attrition bias. Socio-economic differences between OMT group and non-user group might have contributed to over-estimation of group differences.	High
**Arter et al., 2021 Longitudinal outcomes of children exposed to opioids in-utero: A systematic review**	To summarise evidence of long-term outcomes of children, 2 years or older, exposed to opioids in utero.	43 studies 1979 to 2019. Inclusion criteria: empirical studies measuring health or developmental outcomes in children aged 2-18 years; Exposure to 'drugs of addiction', opioid agonists or partial agonists used in MAT, or prescribed opioid analgesics.	Global (all included studies from US, Israel or Western Europe)	No	**Overall development.** Overall development using Bayley scales (n=8) show mixed results (note results are poorly described): 5/8 showed an association between opioid exposure and poorer outcomes overall. Authors suggested that this seemed to be aligned with version used with older versions showing more mixed results. Studies using newer versions consistently demonstrated poorer results for opioid exposed.<br>**Cognitive skills and language development.** Cognitive: mixed results – 3/5 studies using General Cognitive Index subscale of the McCarthy Scales of children's abilities demonstrated poorer scores for opioid exposed children.<br>Language: 5 studies using 4 instruments – 4 found poorer development for opioid exposed with one showing no difference.<br>**Motor skills.** 3 /4 studies using the Motor subscale of the McCarthy Scales of children's abilities found significantly worse scores for exposed children.<br>**Externalising problems.** Adhd: 3 studies found poorer scores for opioid exposed but 4 studies found no significant difference;<br>Behaviour: 4 studies found increased internalising and externalising behaviours for opioid exposed;<br>**Internalising problems.** 7 studies used six scales - 5 found lower scores for opioid exposed.	Large methodological differences in measures used, population characteristics, control of confounding.	Low numbers within studies - out of 43 included studies, 32 had <100 participants. Only 3 studies reported power analysis or effect sizes. Note that cultural differences may affect outcomes. Causal relationships difficult to ascertain due to sample sizes, heterogenous populations, polydrug use, multiple scales used and lack of/variation in controlling confounders.	High
**Conradt 2019 Prenatal Opioid Exposure: Neurodevelopmental Consequences and Future Research Priorities.**	To review the research on short- and long-term neurodevelopmental outcomes of children with prenatal opioid exposure.	52 studies comprising 50,000 children from 350 sites across the USA. Exposure: prenatal opioid exposure and at least 1 neurodevelopm	United States	No	**Cognitive skills and language development/ Motor skills.** Outcomes within 2 yrs: Prenatal opioid exposure associated with impaired language development, neuromotor and psychomotor development before 24m. Studies that accounted for socioeconomic confounding found no difference in cognitive outcomes between exposed and non-exposed children. One study that revealed that boys, but not girls, exposed to opioids and amphetamines, benzodiazepines, cannabis, and tobacco had significantly lower mental development scores by using the Bayley Scales of Infant Development compared with unexposed infants matched on age and after controlling for caregiver socioeconomic status. No difference for neurodevelopmental outcomes at 4m between children exposed to buprenorphine vs methadone for 1 study: another RCT found significantly lower scores	Highly heterogeneous.	Of studies looking at development prior to 24m, 6/11 failed to account for important confounding or have control group. Highlights many flaws with included studies. Key issues 'plagued with small sample	Medium

Author/ year/ title	Objectives	Included studies	Setting	Meta-analysis	Results	Heterogeneity	Limitations	Quality Score
	focusing only on the human literature to describe how these outcomes may vary as a function of (1) the type opioid to which the child is exposed, (2) whether the child was diagnosed with NAS, and (3) whether adverse neurodevelopmental outcomes are present after controlling for confounding variables.	ental outcome at birth or later in development were examined. No publication date range specified. Findings were separated by developmental period: newborn, infancy, and childhood			for expressive and receptive language for children exposed to buprenorphine (vs methadone) at 12m. Neurodevelopmental findings for children with NOWS vs those exposed to opioids without NOWS were inconsistent. Outcomes beyond 2 yrs: Inconsistent results for cognitive outcomes; some studies found no differences in cognitive outcomes. Other studies found differences in IQ, neurologic performance and language, although not always when controlling for co-variates. 1 study found children exposed to buprenorphine in pregnancy had lower IQ, memory and motor skills (1SD below mean) at age 5-6, although no control group.<br>**Externalising problems.** More consistency in behavioural results: children exposed to methadone have higher levels of aggression (age unclear), and executive functioning skills (age 4). Elevated symptoms of ADHD for those exposed to opioids (age not clear), controlling for socio-demographic factors. Diagnosis of NOWS resulted in lower levels of attention vs. Unexposed children, controlling for sex and age.<br>**Internalising problems.** Children exposed to methadone have higher levels of fear and anxiety (age unclear).<br>**Visual outcomes.** 3 studies on visual outcomes: all at age 4 – children exposed to opioids had lower visual motor/perceptual performance scores, poorer lower left visual eye acuity, controlling for SEC. 1 study found no difference in visual perception but methadone-exposed made fewer goal-directed eye-movements, controlling for maternal education.		sizes', lack of adjustment for confounding/control groups, lack of focus on NOWS. Most focussing on retrospective records reviews.	
**Hemmati et al., 2022. Ophthalmic outcomes in children exposed to opioid maintenance treatment in utero: a systematic review and meta-analysis**	The primary aim is to determine whether there is a causal relationship between in utero opioid exposure and future visual abnormalities.	Inclusion criteria: studies of infants or children aged 0–18 years old, with prenatal exposure to opioids, presence of non-opioid exposed control groups, and measurements of ophthalmic outcomes. 9 papers across 5 studies. 1995 prenatally exposed children were compared to 782,042 non-exposed children. Ages in studies: 13 weeks to 10 years.	Global but studies from Australia, Canada, Norway, Scotland.	Yes	**Visual outcomes.** Most studies conducted have measured visual evoked potentials (VEPs) in this population, which are an effective measure of afferent visual integrity and maturity. Methadone exposed infants who were tested at 4 months displayed significant prolonged VEP latencies compared to non-exposed controls in response to both 48' and 69' chequerboard patterns (SMD=0.728, p < 0.0001, z = 3.62). On the other hand, no differences in VEP latencies were identified between methadone exposed infants who were tested at 36 months and non-exposed controls (SMD=− 0.351, p = 0.22, z = − 1.20). No statistically significant results for buprenorphine vs non-exposed. Methdone exposed had prolonged VEP latencies to checks of 48' compared to buprenorphine exposed infants (SMD=0.510, z = 2.11, p < 0.05). However, no VEP differences between methadone exposed infants and buprenorphine exposed infants were detected in response to 69' checks (SMD=0.22, z = 0.54, p = 0.58). 1 study measured mirror neurone system by measuring gaze arrival at the area of interest using a human versus mechanistic design. The opioid exposed cohort took a longer time to shift gaze in the human condition compared to the controls and no difference in mechanistic. 1 study at 6 months found 40% of their methadone exposed group failed the visual assessment criteria compared to 8% of the controls, with the ophthalmic abnormalities including nystagmus, strabismus, reduced visual acuity and maturity (RR 5.1 (CI 1.3 – 20, p = 0.02)). Same cohort: of the 32 drug-exposed infants who failed the visual assessment, 63% had strabismus, 28% horizontal nystagmus, 56% reduced visual acuity, and 6% delayed visual maturity.	High heterogeneity between the pooled studies was only identified for methadone vs control VEP analyses	Small sample sizes, lack of geographical spread, lack of controlling for confounding all highlighted as issues within the review.	High
**Lee et al. 2020 Neurodevelopmental Outcomes of Children Born to Opioid-Dependent**	To conduct a meta-analysis of development outcomes (cognitive, language, motor, and social	Children aged between 0 to 12 years of age, born to opioid dependent mothers with control group.	Global	Yes	**Cognitive skills and language development.** 9 studies explored cognitive performance: opioid exposed had significantly lower cognition scores than non-exposed infants (SMD= 0.77, 95% CI –1.06 to –0.48); lower psychomotor scores (SMD= 0.52; 95% CI –0.78 to –0.25).	High levels of heterogeneity	Lack of adjustment for environmental confounding, lack of distinguishing between different types of opioids. Timing, frequency and extent	High

Author/ year/ title	Objectives	Included studies	Setting	Meta-analysis	Results	Heterogeneity	Limitations	Quality Score
**Mothers: A Systematic Review and Meta-Analysis**	emotional) for children aged between 0 to 12 years born to opioid dependent mothers, compared with children born to mothers that did not use opioids.	16 studies comprising 11 unique cohorts included. 13 studies in preschool, 3 beyond age 5.			5 studies explored language: opioid exposed children had lower IQ scores (SMD –0.76, 95% CI –1.25 to –0.28), lower expressive languages scores (SMD= -0.65; 95% CI –0.97 to –0.34) and lower receptive language scores (SMD= -0.74; 95% CI –1.12 to –0.36).<br><br>**Externalising problems./ Socio-emotional development.** 4 studies looked at externalising and attention problems, respectively (note 2/4 potentially looked at outcomes beyond preschool). Opioid exposed children had higher levels of externalising behaviours (SMD= 0.42; 95% CI 0.17 to 0.68), and attention problems (SMD= 0.72; 95% CI 0.42 to 1.02) than controls.<br><br>**Internalising problems.** 4 studies looked at internalising problems (note 2/4 potentially looked at outcomes beyond preschool). Opioid exposed children had higher levels of internalising behaviours (SMD= 0.66; 95% CI 0.32 to 1.00)		of opioid use not considered. Wide range of follow-up rates/attrition.	
**Monnelly et al., 2019 Childhood neurodevelopment after prescription of maintenance methadone for opioid dependency in pregnancy: a systematic review and meta-analysis.**	To review evidence around the neurodevelopmental outcomes for children born to mothers that were prescribed methadone during pregnancy.	Children 0-18 included. 41 studies identified (1441 methadone-exposed children and 842 unexposed). 8 studies amenable to meta-analysis.	Global	Yes	**Cognitive skills and language development/ Motor skills.** For Mental Developmental Index (MDI) (5 studies) and Psychomotor Development Index (PDI) (4 studies) at 6 months differences between exposed and non-exposed were marginal and non-significant. At 2 years 7 studies reported MDI: the weighted mean difference (WMD) between children exposed to methadone during pregnancy and those who were unexposed was -4.3 (95% CI -7.24 to –1.63). Four studies reported PDI at age 2: WMD was -5.42 (95% CI -10.55 to –0.28).<br><br>1 study reported poorer fine motor skills, less attentiveness and lower motor scores on Bayley at 9m but no difference in cognitive scores. Further study reported no difference in free play at 24m.<br><br>6 studies reported no difference in cognitive outcomes beyond age 2. 4 studies reported lower cognitive performance for opioid-exposed children at ages 2.5-5.Two studies reported lower language skills at ages 3 and 4.<br><br>**Externalising problems.** 6/7 studies reported high levels of behavioural problems for methadone exposed children.<br><br>**Visual outcomes.** 12 studies reported visual outcomes – 5 of which reported Visual Evoked Potentials (VEPs): studies found poorer outcomes for methadone exposed children at 4-6 months, however one study found no difference when children were followed up at age 3. A further 6 studies described other poorer visual outcomes for children exposed to methadone, however all were said to be of poor quality. Visual outcomes were also poor, with increased reports of nystagmus and strabismus	High levels of heterogeneity in terms of outcome measures. All babies exposed to methadone, although dose and frequency not normally considered.	Several studies had a high risk of bias. Only 1 study deemed to be of good quality.	High
**Nelson et al., 2020 Cognitive Outcomes of Young Children After Prenatal Exposure to Medications for Opioid Use Disorder: A Systematic Review and Meta-analysis.**	To determine the consistency of findings regarding the association of prenatal exposure to methadone and buprenorphine with early childhood cognitive developmental when accounting for recruitment imbalances in the included studies.	Inclusion criteria were cohort studies, studies including children aged 1 to 60 months with at least 2 months of prenatal MAT exposure, studies using standardized direct observation	Global	Yes	**Cognitive skills and language development.** Lower cognitive scores for children exposed to MAT during pregnancy compared to the unexposed cohort (pooled SMD, −0.57; 95% CI, −0.93 to −0.21) (16 cohorts from 6 countries). When taking into account prenatal tobacco exposure (4 cohorts only), the association between MAT and cognitive scores was no longer statistically significant −0.11 (95% CI, −0.42 to 0.20).	High (low for smoking cohorts)	Mean quality of studies was low. Most studies had poor internal validity, particularly regarding recruitment bias. Many studies fail to take into account confounding factors, such as tobacco use, when examining the association between MAT and neurodevelopment outcomes.	High

Author/ year/ title	Objectives	Included studies	Setting	Meta-analysis	Results	Heterogeneity	Limitations	Quality Score
		testing scales, and studies reporting means and SDs. 27 studies comprising 16 unique cohorts.						
**Rees et al., 2020. Childhood health and educational outcomes after neonatal abstinence syndrome: a systematic review and meta-analysis**	To determine the frequency of adverse health and educational outcomes after Neonatal Abstinence Syndrome compared with outcomes of unexposed children.	Inclusion criteria: 1975 onwards; Children 28 days to 16; NAS exposure and educational or health outcomes. 15 studies identified/6 amenable to meta-analysis (10,907 exposed children and 1,730, 213 unexposed). Age range 0-16, however authors note that age for specific assessments were often not given. Results extracted here were where we could identify they were within the preschool period.	Global	Yes	**Cognitive skills and language development.** 4 studies looked at preschool speech and language problems; 3 of which met our age criteria, and 2 were homogenous enough for meta-analysis:the pooled OR for speech and language impairment following NAS was (OR 2.81, 95% CI 1.82 to 4.33). One study further provided adjusted ORs in which the relationship was maintained: (OR 2.42, 95% CI 1.35 to 4.34). 1 study demonstrated increased levels of Intellectual Disability in children with NAS at ages 1-5 (3.5% vs 1.5%).<br>**Externalising problems/ Internalising problems.** Increased odds of ADHD from 3 studies – pooled OR 3.21, but variation by age and differences in ascertainment. Note 1 study with results at a range of ages was non-significant when looking at the < age 6 results only, 4 studies explored behavioural/emotional difficulties – 3 of which were in children aged <5 years. 2 studies found a higher risk of behavioural or emotional disorders after NAS at age 2-4 (OR 5.31, 95% CI 2.56 to 11.02), and <6 years (OR 2.17, 95% CI 1.35 to 3.11). However the latter was not significant once confounders were controlled for (including demographics, pregnancy and birth complications). A further study found an increased risk of conduct disorder specifically following NAS (OR 2.88, 95% CI 2.37 to 3.5).<br>**Visual outcomes.** 7 studies explored visual outcomes, 5 of which focused on preschoolers. Studies were consistent in demonstrating increased odds of strabismus and nystagmus following NAS (note – pooled odds not included as these include the two studies outwith our criteria).	High levels of heterogeneity in many places (the exception being visual outcomes) - usually relating to a range of measures being used and wide range of ages.	Some studies non-randomised, incomplete reporting of findings, likely unadjusted confounding.	High
**Romanowicz et al., 2019 The effects of parental opioid use on the parent-child relationship and children's development al and behavioral outcomes: a systematic**	To summarise and review evidence on the impact of parental opioid use on parent-child relationships, attachment, child development and behavioural outcomes.	Inclusion criteria: Children aged from 0-16 years of age; studies exploring opioid use and parent-child relationships. 12 studies were included.	Global	No	**Cognitive skills and language development.** 1 study indicated children exposed to opioids had lower intelligence scores, lower social adaptivity scores and were more likely to be developmentally delayed than controls.<br>**Behaviour and attention/hyperactivity problems.** Children born to mothers receiving methadone treatment were more likely to be more hyperactive and had more conduct problems than controls in one study. However, another study of 2 year olds showed no difference in focussed attention between children exposed prenatally to opioids and controls.	Unknown	Most studies either had a control group or controlled for confounding. However, most studies used small sample sizes and the drug use was often not well described. Most studies recrtuied from low SES groups and excluded substance use in high SES groups.	Medium

Author/ year/ title	Objectives	Included studies	Setting	Meta-analysis	Results	Heterogeneity	Limitations	Quality Score
**review of published reports.**								
**Welton 2019 Effects of opioid use in pregnancy on pediatric development and behaviour in children older than age 2: Systematic review.**	To summarise information on effects of opioid use in pregnancy on subsequent pediatric development and behaviour.	Inclusion criteria: Children older than 2 years, English language, focusing on opioid use in pregnancy and developmental/ behavioural outcomes. Excluded studies including alcohol/polydrug use. 19 studies selected.	Global (English language only) - 12/19 set in Scandinvia.		**Cognitive skills and language development.** 9 studies assessed cognitive development in preschool years: cognitive deficits more strongly correlated with SES than drug use. In one study of 33 children age 2, initial differences in 'mental development' were no longer present once environmental risk was controlled for, whilst a further 2 studies found no relationship between opioid exposure and memory deficits once SEC or maternal education was controlled for. 5 studies (4 in preschool period) explored language development and found prenatal opioid exposure to be associated with language delay. One RCT of methadone vs buprenorphine found deficits in language and cognitive development which normalised by age 3. 3 other studies showed ongoing language and verbal skills up to age 6.<br><br>**Motor skills.** 3 studies showed early delay in motor skills that normalised by age 2-3. A further study found motor development remained below normal at ages 5-6. 2 studies at age 4 found delays in fine motor skills, although in one of these studies this association disappeared when confounding was controlled for.<br><br>**Behaviour and attention/hyperactivity problems.** Behavioural and conduct problems were more likely when children were exposed to opioids in pregnancy. One study demonstrated a worsening of attention and hyperactivity by 4.5 years. Being adopted did not appear to change the association. Effects were exacerbated when children were living in low SEC households and for boys.	High – in terms of study participants and range of measures use.	Studies were small – 5/19 had <35 participants.	Low
**Yeoh 2019 Cognitive and Motor Outcomes of Children With Prenatal Opioid Exposure: A Systematic Review and Meta-analysis.**	To determine the association between Prenatal Opioid exposure and neurodevelopmental outcomes in children aged 0-18.	Inclusion: Published cohort studies comparing the results of age-appropriate standardized cognitive and/or motor tests between children with any POE (aged 0-18 years) with drug-free controls were included. 26 cohort studies were included comprising 1455 exposed children and 2982 unexposed. Results were split into 3 age groups: toddler, preschool & school.	Global (English language). All papers extracted were from HIC (11 from US).	Yes	**Cognitive skills and language development.** For infants (0-24m), 13 studies looked at cognitive development. Results for exposed children were lower ($d = -0.52$; 95% CI, -0.74 to -0.31; $P < .001$) than controls. For ages 3-6yrs (13 studies), there was also a lower level of cognitive development for exposed children compared with controls ($d = -0.38$; 95% CI, -0.69 to -0.07; $P < .02$).<br>**Motor skills.** Results for all exposed children under age 6 showed lower levels of motor skills ($d = -0.49$; 95% CI, -0.74 to -0.23; $P < .001$) than those of the controls.<br>Sensitivity analyses for children with NAS, and children removed from their birth parent's care, demonstrated no significant associations in either age group.	High – in terms of measures of opioids and outcomes.	Lack of controlling for confounding in some studies.	High
