# Supplementary material for: Impact of exposure to opioids in pregnancy on offspring developmental outcomes in the preschool years: an umbrella review
Source: BMJ Paediatr Open. 2025 Jan 9;9(1):e003058. doi: 10.1136/bmjpo-2024-003058 (PMC11749434; doi:10.1136/bmjpo-2024-003058)
Supplement: Supplementary file 4 [file bmjpo-2024-003058.reviewer_comments.pdf]

PEER REVIEW HISTORY

BMJ Paediatrics Open publishes all reviews undertaken for accepted manuscripts. Reviewers are asked to complete a checklist review form and are provided with free text boxes to elaborate on their assessment. These free text comments are reproduced below.

ARTICLE DETAILS

**TITLE (PROVISIONAL)**	The impact of exposure to opioids in pregnancy on offspring developmental outcomes in the pre-school years: an Umbrella Review
**AUTHORS**	Robertson, Senga; Hughes, Thomas; Boardman, James; McFadden, Anne; Whittaker, Anne; Marryat, Louise

VERSION 1 - REVIEW

**REVIEWER NAME**	***Hannah Uebel***
**REVIEWER AFFILIATION**	University of New South Wales, School of Women's and Children's Health
**REVIEWER CONFLICT OF INTEREST**	Not applicable
**DATE REVIEW RETURNED**	10-Oct-2024

**GENERAL COMMENTS**	Can word counts be provided for all sections of the article?<br><br>Abstract: can citations to be included for statements made?<br><br>Methods: what was the definition for "opioid exposure"? Did you include reviews relating to prescribed opioid medications? What were the key words used to find articles? You specify these things later on but may be relevant to include more information in the abstract also?<br><br>Conclusion: Line 21 of conclusion – "Robust research, with larger sample sizes and adequate accounting for confounding, is needed to provide accurate information for both women and practitioners.". Are you referring to women of chid bearing age here? I agree this is important, but I would argue also extremely important to recognise the need for more information to guide policy and ensure appropriate funding, support and follow-up is in place?<br><br>What is already known: 2nd dot point, Lines 11-16. You reference multiple systemic reviews exploring the association between prenatal opioid exposure and adverse early childhood outcomes across different domains, and then state that "no systematic review of evidence across developmental domains has been conducted?". This seems contradictory. Additionally, does "different domains" refer to different developmental domains? This needs to be specified and kept consistent.<br><br>What this study adds: It's tricky to interpret the lack of evidence regarding cognitive development, as formal cognitive testing is not usually completed until school age.

	How this study might affect research, practice or policy: 1st dot point, line 44 – “social care practitioners”. This may be regional differences in terminology, I would usually refer to this as “social worker” or “psychosocial practitioners”.<br>You have mentioned supports in school, but maybe also relevant to mention the need for further studies to assess the outcomes of children at school and look at cognitive testing results.<br><br>Introduction:<br>3rd line (line 11-12) “neonatal outcomes: findings demonstrate…” – should this be a ; or fullstop and new sentence?<br>You should also refer more directly to toxic effects on the developing brain, and the complex interaction between genetic influences and environmental factors.<br>Line 23-28: sentence does not make sense and needs rewording – “The evidence that does exist suggests later adverse outcomes across a range of domains, including education, behavioural difficulties, social, and poor health outcomes”<br>Line 35. Page 7: incorrect spelling, “focussing” should be “focusing”<br>Line 46-47: provide citation please, which policy makers are interested?<br>Line 51 onwards (“with a range of factors, including experiencing adversity in the early years, living in poverty, low parental education levels, child neglect and/or maltreatment, domestic abuse, parental mental-health issues, and removal from the birth parent into social care”): The list are adversities, consider amending to “with a range of adverse childhood experiences including living in poverty etc…”<br><br>Methods:<br>Search strategy – you did not use the keyword “Neonatal abstinence syndrome” – was there a reason behind this?<br><br>Public and Patient Involvement – This could be neatened and made more scientific in writing style.<br><br>Results: Your end number of papers included was small (11). Did you go to efforts to get the three text articles that were not available?<br>Quality appraisal – your overall rating of the quality was “high” for 8/11 papers included, yet your conclusion is that the quality of evidence is low?<br>Table 4, “*Results were extracted up to 6 years only.” In the table there is no * next to the Yeoh paper age range however this is written in brackets?<br>Can you provide information on what the outcome measures were? Ie, what assessments were used? Were they utilising standardised assessments, such as Baileys or WPPSI?<br>Statement line 20-21, page 21: “No differences were found between children exposed to illicit vs. prescription opioids”. Weren’t prescription opioid studies excluded from this review?<br>Page 21, externalising problems: Although there is evidence regarding ADHD, it is inaccurate to attempt to interpret the outcomes of ADHD in your study if you only include children up to age 5. Most ADHD diagnoses come after a child starts primary school, and there is a hesitation from many clinicians to make a diagnosis prior to this age. This is something that should be explored further in the discussion.<br>Page 22, Internalising problems: I would argue that internalising problems are quite uncommon in the toddler age group, as signs of

	anxiety are often non specific and difficult for parents and clinicians to recognise in this young age.<br><br>Discussion<br>Statement "there appears to be fairly strong" evidence between exposure to opioids and visual outcomes, motor skills, externalising problems, and language difficulties; can this be strengthened or made more committal?<br>When discussing cognitive results, does evidence suggest that opioid exposed children "catch up" – I think the nature of "cognitive testing" in preschool age children is somewhat limited by the gross nature of testing and the increasing difficulty in follow-up as children get older, meaner older cohorts often have smaller cohorts and the statistical significance is likely to diminish. This conclusion is somewhat illogical, as language skills are one of the greatest predictors of cognitive ability. Certainly studies looking at longterm educational outcomes of children with prenatal drug exposure have shown that opioid-exposed children are significantly behind their peers, even when adjusted for confounding factors.<br>There is no exploration in your discussion of other factors that influence developmental and cognitive outcomes. This is critical.<br>Limitations, page 23, line 54 – incorrect spelling of word "focusing".<br>In limitations, you need to discuss the reasons as to why the data is limited, the difficulties in longterm follow-up for this vulnerable and chaotic population, the under reporting of maternal drug use, the increased experience of adverse childhood events in this population group, and the inability of many studies to control for this extensive list of confounders that can influence developmental and cognitive outcomes.<br>Conclusion, page 24:<br>Line 14-16 – internalising behaviours is not listed in the outcomes measured<br>Line 18 onwards – sentence is far too long and needs revising. ("Having the evidence on the impact of exposure to opioids in pregnancy on child developmental outcomes is important for health and social care practitioners, as well as women who used opioids, to enable them to understand the likely impact of opioids on the child, beyond the postnatal period, and to enable children to be more closely monitored and for additional support to be put in place for children and families where required")<br>Conclusion is too wordy and needs to be tightened. Some of this information is more appropriate for the discussion.<br><br>Search strategy: is there a reason terms such as "prenatal drug exposure" and "neonatal abstinence syndrome" were not utilised in the search?<br>PsychInfo: Why does the search strategy include Year 2023 TO 2024 (you have stated that you searched from 2016 onwards)

**REVIEWER NAME**	***Ju Lee Oei***
**REVIEWER AFFILIATION**	Royal Hospital for Women, Department of Newborn Care
**REVIEWER CONFLICT OF INTEREST**	Not applicable
**DATE REVIEW RETURNED**	20-Oct-2024

**GENERAL COMMENTS**	Thank you for the opportunity to review this review of reviews. It is a very much needed project.

	I hope that this review will be helpful<br><br>1. Abstract - would appreciate more information for results<br>2. What is known section - suggest more definitive recommendations. Currently it is on the vein of "we are concerned but not enough is known". What can we do as clinicians, researchers and policy makers?<br>3. Introduction - please be more specific about previous work. What were the previous reviews? summarise their findings. Why should this work be done? Why stop at 5 years?<br>4. Methods - no issues<br>5. Results: what does "wrong study design" mean?<br>6. Discussion: well written but I would suggest stronger commitment to recommendations - do or don't the authors recommend that follow-up be provided to children with prenatal opioid exposure, what constitutes a level of exposure that would cause concern and how would follow-up and support be implemented in the general population, considering the massive numbers of children in this community?<br><br>Minor<br>line 22, Page 24 - findings not finding

VERSION 1 – AUTHOR RESPONSE

Reviewer: 1
Dr. Hannah Uebel, University of New South Wales, Sydney Children's Hospital Randwick

1. Can word counts be provided for all sections of the article?	Abstract: 299<br><br>Intro: 630<br><br>Methods: 838<br><br>Results: 1638<br><br>Discussion: 1218<br><br>Conclusions: 192<br><br>Total: 4516<br><br>Although we have tried to keep the additional information in response to reviewer suggestions as concise as possible, we are now c.500 words over the word count. We have asked the editor if this is allowed.
2. Abstract: can citations to be included for statements made?	The journal style does not allow for references in the abstract, so we have left this as it stands.
3. Abstract/Methods: what was the definition for “opioid exposure”? Did you include reviews	Lines 48-49: We have added a definition (‘(illicit (e.g. heroin/fentanyl) opioids, and prescribed

relating to prescribed opioid medications? What were the key words used to find articles? You specify these things later on but may be relevant to include more information in the abstract also?	treatments for opioid dependence)'). We weren't able to add key words (and had to cut some words in the conclusion) to keep within the 300 word allowance.
4. Conclusion: Line 21 of conclusion – "Robust research, with larger sample sizes and adequate accounting for confounding, is needed to provide accurate information for both women and practitioners.". Are you referring to women of chid bearing age here? I agree this is important, but I would argue also extremely important to recognise the need for more information to guide policy and ensure appropriate funding, support and follow-up is in place?	Yes, we are referring to women of child-bearing age – we have clarified this in Line 68.<br><br>We agree with your point about guiding policy/funding decisions - this has been added in Lines 69-70.
5. What is already known: 2nd dot point, Lines 11-16. You reference multiple systemic reviews exploring the association between prenatal opioid exposure and adverse early childhood outcomes across different domains, and then state that "no systematic review of evidence across developmental domains has been conducted?". This seems contradictory. Additionally, does "different domains" refer to different developmental domains? This needs to be specified and kept consistent.	Lines 75-78: We have made this clearer.
6. What this study adds: It's tricky to interpret the lack of evidence regarding cognitive development, as formal cognitive testing is not usually completed until school age.	This is an interesting point. I agree that more formal testing doesn't tend to be conducted until at school age, and I have added a bit into Lines 84-82 to clarify this. I would suggest that we are good at doing this in the preschool years in our birth cohorts, however we are not good at capturing this particular population of kids in our birth cohorts.
7. How this study might affect research, practice or policy: 1st dot point, line 44 – "social care practitioners". This may be regional differences in terminology, I would usually refer to this as "social worker" or "psychosocial practitioners". You have mentioned supports in school, but maybe also relevant to mention the need for further studies to assess the outcomes of children at school and look at cognitive testing results.	Line 90 – this has been amended.<br><br>We have added in further points around implications for research and practice/policy in Lines 93-101
8. Introduction:<br>3rd line (line 11-12) "neonatal outcomes:	Line 105: we have changed this to a full stop.

findings demonstrate…" – should this be a ; or fullstop and new sentence?	
9. You should also refer more directly to toxic effects on the developing brain, and the complex interaction between genetic influences and environmental factors.	Lines 130-134: we have added in a little more on the genetic influences. We have not added further to the brief description (Lines 126-129) of the impact on the brain due to space considerations.
10. Line 23-28: sentence does not make sense and needs rewording – "The evidence that does exist suggests later adverse outcomes across a range of domains, including education, behavioural difficulties, social, and poor health outcomes"	Lines 110-112: We have made this clearer.
11. Line 35. Page 7: incorrect spelling, "focussing" should be "focusing"	We believe this is different in American English and British English– we have kept the British English spelling as BMJ is a British based journal.
12. Line 46-47: provide citation please, which policy makers are interested?	Line 120: we have added an example here.
13. Line 51 onwards ("with a range of factors, including experiencing adversity in the early years, living in poverty, low parental education levels, child neglect and/or maltreatment, domestic abuse, parental mental-health issues, and removal from the birth parent into social care"): The list are adversities, consider amending to "with a range of adverse childhood experiences including living in poverty etc…"	Lines 122-123: this has been changed.
14. Methods:<br>Search strategy – you did not use the keyword "Neonatal abstinence syndrome" – was there a reason behind this?	We discussed this with regards to the search strategy, however it brought up a lot of papers with outcomes of NAS/NOWS rather than exposures so we removed it. We were also confident from our investigations that any NAS/NOWS relating to opioids mentioned the substance in the abstract. We have noted that we accidentally left in NOWS in the criteria (Table 1) – this has now been removed.
15. Public and Patient Involvement – This could be neatened and made more scientific in writing style.	Lines 238-243: we have adjusted this to make it neater and more scientific in writing.
16. Results: Your end number of papers included was small (11). Did you go to efforts to get the three text articles that were not available?	For all three papers our librarian attempted to obtain the full text through tools at their disposal, and we attempted to find and contact authors but without success. We have not included this information in the body of the text due to word

	constraints. Line 250: we have added ‘despite efforts to locate them’.
17. Quality appraisal – your overall rating of the quality was “high” for 8/11 papers included, yet your conclusion is that the quality of evidence is low?	Our quality appraisal was of the systematic reviews themselves rather than the original papers included in those systematic reviews. It is these original papers which are of low quality, not the systematic reviews. We have tried to make clear throughout when we are talking about individual papers/studies and the reviews. We have made this clearer e.g. Line 266.
18. Table 4, “*Results were extracted up to 6 years only.” In the table there is no * next to the Yeoh paper age range however this is written in brackets?	This was inconsistent. We have now added a * next to Yeoh in line with the other reviews for whom we extracted results for a limited age range.
19. Can you provide information on what the outcome measures were? Ie, what assessments were used? Were they utilising standardised assessments, such as Baileys or WPPSI?	We chose not to summarise measures used in the 478 component studies, preferring readers to access their full descriptions in there original works, however we have added some contextual information in the cognitive assessment section to give a flavour of the heterogeneity – Lines 307-310.
20. Statement line 20-21, page 21: “No differences were found between children exposed to illicit vs. prescription opioids”. Weren’t prescription opioid studies excluded from this review?	We excluded prescription opioids for pain relief. Methadone and Buprenorphine are primarily prescribed. Line 335: we have made this clearer. We have also made this clearer in Table 2.
21. Page 21, externalising problems: Although there is evidence regarding ADHD, it is inaccurate to attempt to interpret the outcomes of ADHD in your study if you only include children up to age 5. Most ADHD diagnoses come after a child starts primary school, and there is a hesitation from many clinicians to make a diagnosis prior to this age. This is something that should be explored further in the discussion.	We have added some contextual information into the discussion: lines 390-393.
22. Page 22, Internalising problems: I would argue that internalising problems are quite uncommon in the toddler age group, as signs of anxiety are often non specific and difficult for parents and clinicians to recognise in this young age.	We have a discussion point on this in lines 414-417.
23. Discussion<br>Statement “there appears to be fairly strong” evidence between exposure to opioids and visual outcomes, motor skills, externalising	We have added some detail to this in Lines 384-386.

problems, and language difficulties; can this be strengthened or made more committal?	
24. When discussing cognitive results, does evidence suggest that opioid exposed children “catch up” – I think the nature of “cognitive testing” in preschool age children is somewhat limited by the gross nature of testing and the increasing difficulty in follow-up as children get older, meaner older cohorts often have smaller cohorts and the statistical significance is likely to diminish. This conclusion is somewhat illogical, as language skills are one of the greatest predictors of cognitive ability. Certainly studies looking at longterm educational outcomes of children with prenatal drug exposure have shown that opioid-exposed children are significantly behind their peers, even when adjusted for confounding factors.<br>There is no exploration in your discussion of other factors that influence developmental and cognitive outcomes. This is critical.	We have done 3 things in response to the areas raised here:<br>1. We have made language around the implications of these findings more tentative (Line 397)<br>2. We have added caveats around the findings and raised the evidence around language and cognitive/educational outcomes (Lines 405-412)<br>3. We have added a section about other factors and child development/ the importance of controlling for confounding in the discussion (Lines 426-433)
25. Limitations, page 23, line 54 – incorrect spelling of word “focusing”.	Please see response to point 11.
26. In limitations, you need to discuss the reasons as to why the data is limited, the difficulties in longterm follow-up for this vulnerable and chaotic population, the under reporting of maternal drug use, the increased experience of adverse childhood events in this population group, and the inability of many studies to control for this extensive list of confounders that can influence developmental and cognitive outcomes.	Lines 458-463 – we have added more into the limitations here.
27. Conclusion, page 24:<br>Line 14-16 – internalising behaviours is not listed in the outcomes measured	We hadn’t mentioned this at this point as we were just highlighting the domains with firmer conclusions. We have added in mentions of internalising behaviours and cognitive development in Lines 497-499.
28. Line 18 onwards – sentence is far too long and needs revising. (“Having the evidence on the impact of exposure to opioids in pregnancy on child developmental outcomes is important for health and social care practitioners, as well as women who used opioids, to enable them to understand the likely impact of opioids on the child, beyond the postnatal period, and to enable children to be more closely monitored	Lines 477-482 – this has been split up and made more concise.

and for additional support to be put in place for children and families where required”)	
29. Conclusion is too wordy and needs to be tightened. Some of this information is more appropriate for the discussion.	We have moved some of the information into the conclusions.
30. Search strategy: is there a reason terms such as “prenatal drug exposure” and “neonatal abstinence syndrome” were not utilised in the search?	See point 14 re. NAS/NOWS.<br><br>With regards to ‘prenatal drug exposure’ we did not use this term as it is very broad and we were concerned that general terms around drugs or substances would include papers on other drugs used in pregnancies. We excluded many studies due to other drugs being included, even with the tighter search strategy.
31. PsychInfo: Why does the search strategy include Year 2023 TO 2024 (you have stated that you searched from 2016 onwards)	This was a typo and has been corrected.

Reviewer: 2
Dr. Ju Lee Oei, Royal Hospital for Women, University of New South Wales

32. Abstract - would appreciate more information for results	We have added some additional results in (Lines 54-60). Beyond this we are finding it difficult to add further detail to the results within the 300 word limit.
33. What is known section - suggest more definitive recommendations. Currently it is on the vein of "we are concerned but not enough is known". What can we do as clinicians, researchers and policy makers?	Lines 95-101 – we have added some additional suggestions.
34. Introduction - please be more specific about previous work. What were the previous reviews? summarise their findings. Why should this work be done? Why stop at 5 years?	We haven’t summarised the findings of the previous reviews as this comprises the bulk of this paper. We have expanded on why this work should be done and why the focus on this age period in Lines 151-153.
35. Methods - no issues	-
36. Results: what does "wrong study design" mean?	We have added an example in Line 253 – this was mainly reviews that were not systematic, i.e. narrative reviews or overviews of the evidence for clinicians.
37. Discussion: well written but I would suggest stronger commitment to recommendations - do or don't the authors recommend that follow-up be provided to children with prenatal opioid exposure, what constitutes a level of exposure	Overall we are reluctant to provide stronger recommendations given the overall weakness of the evidence. We have added a recommendation in about increasing resource

that would cause concern and how would follow-up and support be implemented in the general population, considering the massive numbers of children in this community?	for universal services such as health visiting in Lines 443-447.
38. Minor - line 22, Page 24 - findings not finding	Line 424 – this has been corrected.
